# A qualitative study exploring the experiences of advanced clinical practitioner training in emergency care in the South West of England, United Kingdom

**DOI:** 10.1136/emermed-2024-214016

**Published:** 2024-10-15

**Authors:** Suzanne Ablard, Maxine Kuczawski, Colin O'Keeffe, Fiona C Sampson, Jedidah Mould, Suzanne M Mason

**Affiliations:** 1 The School of Medicine and Population Health, The University of Sheffield, Sheffield, UK; 2 Division of Population Health, Health Services Research, and Primary Care, The University of Manchester, Manchester, UK

**Keywords:** advanced practitioner, emergency department, teaching

## Abstract

**Background:**

Attempting to improve emergency care (EC) advanced clinical practitioner (ACP) training, Health Education England (HEE) South West (SW) implemented a pilot, whereby emergency departments (ED) were provided with enhanced funding and support to help ED consultants deliver teaching and supervision to EC ACPs to ensure more timely completion of EC ACP training compared with previous cohorts training in the region.

We explored the experiences of trainee EC ACPs and consultant EC ACP leads working in EDs, which had implemented the new regional pilot.

**Methods:**

We used a qualitative design to conduct semi-structured interviews with trainee EC ACPs and consultant EC ACP leads across five EDs that had implemented the HEE SW pilot. Interview data were analysed thematically.

**Results:**

Twenty-five people were interviewed. We identified four themes: (1) the master’s in advanced practice could be better aligned with the Royal College of Emergency Medicine credentialling e-portfolio; (2) EC ACP training needs some flexibility to reflect the individual—‘one size does not fit all’; (3) supervision and teaching were recognised as important but requires significant staff capacity that is impacted by external pressures and (4) unclear role expectations and responsibilities hinder role transition and impact role identity.

It was notable that EC ACPs primarily spoke about the development of their clinical skills both academically and within the workplace, despite there being other skills mentioned in the multiprofessional framework for advanced practice (leadership and management, education and research).

**Conclusion:**

A clear supervision structure with protected time allocated for teaching and assessment of clinical skills within the ED is essential to facilitate trainee EC ACP progression. However, increasing demands on EDs make this a challenging goal to achieve. Role identity issues continue to persist despite the introduction of new guidance designed to provide more clarity around the ACP role.

**WHAT IS ALREADY KNOWN ON THIS TOPIC**

- Advanced clinical practitioners (ACPs) in the UK are experienced healthcare professionals who come from any regulated healthcare professional background (eg, nurses, paramedics, physiotherapists, pharmacists).
- There is international evidence these roles improve both service outcomes and quality of patient care.
- Historically, an absence of clear role definitions and training routes for ACPs have resulted in these roles being introduced in an ad hoc manner in clinical settings, hampering efforts to develop a distinct and complementary role.

**WHAT THIS STUDY ADDS**

- EC ACP training and supervision are compromised by workload pressures in emergency departments (EDs) limiting training opportunities. However, ring fenced time for education delivery from senior ED clinicians as in an ‘Educator Day model’ has the potential to support completion of Royal College of Emergency Medicine (RCEM) e-portfolio requirements for trainee EC ACPs.
- Respondents felt that decisions about RCEM credentialling targets could be made on a case-by-case basis, so that individual experience at training outset and personal circumstances (eg, caring responsibilities, illness) could be considered. This was in preference to a ‘one size fits all’ approach to training deadlines.
- There is a perceived lack of synergy between the master’s in advanced practice (which is not specialty specific—the same modules are taught to all ACPs regardless of clinical specialty) and RCEM credentialling, with the former being described as not clinically focused enough to support EC ACPs in the clinical elements of their training.
- While trainee EC ACPs discussed increasing their skills regarding the clinical pillar of the multiprofessional framework, the other three pillars (leadership and management, education, and research) received less attention.
- Trainee EC ACPs continue to struggle to define the ACP role and where it fits within multidisciplinary teams within the ED.

**HOW THIS STUDY MIGHT AFFECT RESEARCH, PRACTICE OR POLICY**

- Overwhelming pressures on ED services understandably means providing clinical training and supervision to ACPs (as well as for other trainees, such as junior doctors) in the ED environment is challenging. However, providing senior ED clinicians with protected time for education delivery could help overcome some of these challenges.
- Role identity issues continue to persist despite the introduction of the multiprofessional framework, which was designed to create a shared understanding of what advanced level practice entails and how ACPs can be deployed to deliver better patient care.

## Introduction

 Advanced practitioner is an example of an innovative workforce role that has been implemented both in the UK and internationally.^1 2^ These roles have been developed to help address demand pressures and workforce capacity challenges in healthcare, including in emergency care. In the UK, advanced clinical practitioners (ACPs) are experienced healthcare professionals (eg, nurses, paramedics, pharmacists, physiotherapists and occupational therapists) who undertake further education (master’s degree) and extended clinical training to develop the knowledge and skills to independently assess, diagnose and treat patients in a range of clinical settings.^1^ These roles have been shown to improve both service outcomes and quality of patient care.^3 4^ Once qualified ACPs primarily sit on the junior doctor tier (eg, FY2) of medical rotas, but some more experienced ACPs may sit in the middle grade tier alongside specialty trainees.

Historically there has been an absence of clear role definitions and standardised training routes for ACPs, resulting in these roles being introduced in an ad hoc manner in clinical settings, often motivated by the need to fill service gaps, hampering efforts to develop a distinct and complementary role alongside medical staff.^5^ Attempting to tackle this variation, in 2017, the multiprofessional framework was introduced to provide clearer and more consistent guidance on the ACP role and training routes in the UK NHS. According to this framework:

Advanced clinical practice is delivered by experienced, registered health and care practitioners. It is a level of practice characterised by a high degree of autonomy and complex decision making. This is underpinned by a master’s level award or equivalent that encompasses the four pillars of: clinical practice; leadership and management; education; and research, with demonstration of core capabilities and area specific competence.^6^

The multiprofessional framework is the first time that a national framework defining advanced practice has existed in England. It aims to create a shared understanding of what advanced level practice entails and includes an overview of how ACPs can be deployed to deliver better patient care.

In 2015, The Royal College of Emergency Medicine (RCEM) in collaboration with Health Education England (HEE) introduced an emergency care ACP (EC ACP) credentialling process. The credentialing process is a mechanism whereby trainee and established ACPs present evidence of their achievements and competencies to a panel of fellows of the RCEM, and credentialled EC ACPs, who will decide whether the EC ACP has met the standard of working at a similar level to middle-grade medical staff in an emergency department (ED).^7^ The information presented to the panel includes evidence showing they have completed the MSc in advanced practice and RCEM e-portfolio. The RCEM e-portfolio is an official online platform where trainee EC ACPs can upload evidence of their clinical and educational achievements as they progress through their training. The long-term goal is to ensure emergency care produces a workforce consisting of qualified, credentialed EC ACPs.

In 2019 HHE South West (HEE SW) began a pilot of a regional training initiative in five EDs in the South West of England to train a cohort of EC ACPs. As well as implementing the RCEM credentialling process, the regional pilot provided enhanced funding to enable trainee supernumerary working (ie, EC ACP trainees’ time is funded for learning activities, without being counted in the workforce for delivery of the services); access to specialty wide placements (anaesthetics; ICU; acute medicine); regional peer support to create a community of practice for trainees and trainers; regional training events and annual reviews of training. The aim of the pilot was to see whether the enhanced funding, support and guidance provided by HEE SW creates an improved training environment for EC ACPs as well as ensuring more timely completion of training compared with previous EC ACP cohorts training in EDs in the region.

There is currently limited understanding about what impact these new initiatives have had on the training experiences of EC ACPs. We aimed to identify what impact the new regional pilot had on the experiences of trainee EC ACPs and consultant EC ACP leads working in EDs, which had implemented the pilot as well as assessing how EC ACP training could be further improved in the future, with the goal of informing future models of education and support for EC ACPs.

## Methods

### Design and setting

We used a qualitative design to conduct semi-structured interviews across five EDs in South West England, United Kingdom that had implemented the HEE SW pilot. Prior to the implementation of the pilot, the EDs had employed trainee EC ACPs in small numbers, utilising the NHS Apprenticeship Scheme funding to support course fees only; no other additional funding support for trainees or supervisors was available.

We aimed to understand the perspectives of three groups of participants about ACP training: (1) consultant EC ACP leads (provide overall leadership for the clinical training, development and supervision of the ACP team within the ED), (2) pilot EC ACPs (trainee EC ACPS participating in the HEE SW pilot who started training between September 2019 and September 2020) and (3) non-pilot EC-ACPs (those who were undertaking conventional ACP training from 2016 onwards).

### Sampling and recruitment

HEE SW provided contact details for consultant EC ACP leads and pilot EC ACPs for each of the participating EDs. Consultant EC ACP leads acted as gatekeepers in identifying non-pilot EC ACPs who worked in participating EDs.

Eligible participants were sent a study pack via email, which included a cover letter, information sheet and consent form. On completion of the consent form, a member of the study team contacted the participant to arrange a time and date for the interview.

### Data collection

We designed a semi-structured interview guide in collaboration with colleagues working at HEE SW, who helped us identify all the different components of EC ACP training, so that we could explore these during the interviews. The interview guide was adapted for each participant group. Interviews with pilot and non-pilot EC ACPs explored their experiences of undertaking ACP training (eg, support and supervision, educational opportunities, peer support and support from other colleagues, work placements), including ideas about how ACP training could be improved in the future. Interviews with consultant EC ACP leads covered the barriers and enablers encountered with regards to delivering EC ACP training within the ED, supervising trainee EC ACPs and any changes they would like to make to the way ACPs are trained (see onlinesupplemental files 1  2)

Interview schedules were piloted on a trainee EC ACP and consultant EC ACP lead in a hospital site not involved in the pilot. No significant changes to the interview schedule were suggested. Due to the COVID-19 pandemic, interviews were conducted online (using Google Meet) or over the telephone by one author (SA) who is an experienced qualitative researcher with no clinical background or experience. Interviews were audio recorded using an encrypted voice recorder. On completion of the interview, participants received a £30 shopping voucher to thank them for their time.

### Analysis

Interviews were transcribed verbatim and analysed using thematic analysis following the stages described by Braun and Clarke.^8^ Transcripts were read and checked by one member of the team (SA) to gain familiarisation with the data and initial codes generated. The team then moved on to identifying themes, and reviewing, defining and naming themes. Data were compared across interviews to look for similarities and differences between them. NVivo V.12.0^9^ was used to help structure the analysis.

### Ethical considerations

The School of Medicine and Population Health Ethics Committee based at the University of Sheffield granted ethical approval for the study (Ref 037842).

## Results

We recruited 25 people across 5 sites: 13 pilot EC ACPs, 5 non-pilot EC ACPs and 5 consultant EC ACP leads. At the time of the evaluation, 14 EC ACPs were participating in the pilot. Only 1 pilot EC ACP declined to participate because they withdrew from EC ACP training. Each Consultant EC ACP lead was asked to identify 2 non-pilot EC ACPs in their ED department (n=10) who might be interested in participating in the evaluation. Of the 10 non-pilot EC ACPs invited to participate, 5 accepted the invitation. Interviews took place between May 2021 and December 2021. Table 1 provides an overview of the demographics and professional backgrounds of trainee EC ACPs.

**Table 1:** Demographics and professional background

	Pilot EC ACPs, n (%)	Non-pilot EC ACPs n (%)
**Total**	**13**	**5**
Gender		
Male	5 (38)	2 (40)
Female	8 (62)	3 (60)
Age		
25–34	2 (15)	0 (0)
35–44	10 (76)	3 (60)
45–54	1 (7)	2 (40)
Ethnicity		
White	13 (100)	5 (100)
Baseline profession		
Nurse	6 (46)	4 (80)
Paramedic	7 (54)	1 (20)
Year EC ACP started training		
2016	0 (0)	1 (20)
2017	0 (0)	1 (20)
2018	0 (0)	2 (40)
2019	4 (31)	0 (0)
2020	9 (69)	1 (20)

ACPadvanced clinical practitionerECemergency care

### Overarching themes

Table 2 provides an overview of the themes and subthemes that were identified.

**Table 2:** Themes and subthemes

Theme	Subtheme
The master’s in advanced practice could be better aligned with the RCEM credentialing e-portfolio	Due to previous experience and knowledge, trainee EC ACPs can assess/diagnose through recognising patterns in presentations, however, they lack the medical/pathology knowledge to underpin this pattern recognition. This is problematic and can impact confidence. Placements provided valuable work-based learning opportunities. The Masters in Advanced Practice does not appear to provide the complete clinical knowledge required to underpin the ACP role in emergency care. Knowledge sharing by medical professionals could be beneficial and promote greater synergy between the academic and clinical elements of the trainee EC ACP training.
EC ACP training needs some flexibility to reflect the individual—‘one size does not fit all’	Experience, knowledge and skills are highly individualised at the start of training (partly due to ACPs being recruited from a variety of healthcare professional backgrounds), potentially impacting training progression. Workload pressures during training are perceived as significantly higher if the Masters in Advanced Practice is undertaken simultaneously with the RCEM credentialling.
Supervision and teaching requires significant staff capacity that is often impacted by external pressures	Trainee EC ACPs valued the overall support and supervision received from medical colleagues and other EC ACPs. Informal and unplanned interactions contributed significantly to workplace-based learning, providing opportunities for constructive feedback and a supportive learning experience. Frustration was reported when access to, and capacity of senior clinical staff was limited hindering workplace-based assessments. Consultant EC ACP Leads acknowledged challenges in trying to provide teaching and supervision to trainees, of which trainee EC ACPs were just one group. To help achieve this, some Consultant ED teams had implemented “educator days” (as part of an RCEM educator pilot) enabling protected time to deliver workplace-based training and supervision to all ED trainees (not just EC ACPs).
Unclear role expectations and responsibilities hinder role transition and impact role identity	A strong support network including peers was invaluable. Transitioning from novice to expert senior decision-maker was challenging for all trainee EC ACPs. Ambiguous role expectations and responsibilities exacerbated transitional anxieties.

ACPadvanced clinical practitionerECemergency careRCEMRoyal College of Emergency Medicine

### The master’s in advanced practice could be better aligned with the RCEM credentialing e-portfolio

The data identified what respondents felt were the key elements of EC ACP training, with comparisons often made to the training undertaken by junior doctors.

Trainee EC ACPs and consultant EC ACP leads accepted that trainee EC ACPs have significant clinical experience gained through many years of working in the NHS but that the trainee EC ACP clinical decision-making tended to be driven by pattern recognition. However, trainee EC ACPs lack knowledge about the pathophysiological mechanisms associated with that diagnosis. A consultant EC ACP lead described the moment they realised that learning needs differ between trainee EC ACPs and junior Doctors:

Had this lightbulb conversation about 4 years ago with one of the trainees who’s now a senior ACP and I asked her something about cardiac output and she just looked at me like this, kind of like, “come on you know about the heart, tell me about this” … and I just realised like how little basic science they knew (Consultant EC ACP lead 5)

Trainee EC ACPs described opportunities to increase their clinical knowledge during workplace-based training through supervision by medical colleagues and completion of the RCEM credentialling e-portfolio. The opportunity to attend placements in departments outside the ED (ICU, anaesthetics, acute medicine) was considered a strength of the HEE SW pilot. Many pilot EC ACPs said that, due to the COVID-19 pandemic, these placements had not gone ahead as planned, but those who had attended them described them as valuable and enjoyable.

I think it’s been useful to see what happens further down the line to kind of understand the patient’s journey a bit more (Pilot EC ACP 6)

There were mixed views among trainee EC ACPs about how useful modules on the master’s in advanced practice were to their workplace-based training and completion of the RCEM credentialling e-portfolio. Some modules (eg, physiology and diagnostic reasoning; independent prescribing) were rated highly, whereas others were seen as a tick-box exercise (eg, the leadership module).

The physiology and diagnostic reasoning was a complete mind blowing module, I thought wow that makes sense now, oh yes that’s why we do that…That was a really, really beneficial module. The leadership and innovation is, or was a box-ticking module and I literally took nothing away from that at all (Pilot EC ACP 8)

Some respondents felt the master’s in advanced practice does not fully equip trainee EC ACPs with the knowledge they need to fulfil their role within the ED.

I think there’s a stark difference. I mean, even if you compare us to the physician’s assistants, they’re sort of … very sort of biomedical degree, and I think their understanding of, you know, basic pathophysiology, biochemistry, just the way they’re educated is very different to us… I don’t think the masters in advance practice programmes provide that … they certainly help, but not the, you know, sort of molecular level that doctors train at…. You have to learn that yourself as you go along (Pilot EC ACP 7)

The master’s in advanced practice is supposed to be taught by a mixture of allied health professionals, nurses and doctors, but this is not always the case, with ACPs in this study highlighting a lack of doctors teaching on master’s courses. One participant suggested that medical colleagues should be invited to lecture on the clinical elements of the master’s course to create greater synergy between the academic and workplace-based (clinical) training.

I feel it should be taught by medics, like with a medical degree. It should all be done by doctor’s cos then they teach you in that style. They teach you in a style that doctors have to do because then you won’t have this huge gap with your skills … the structure I need to follow is the medical model. And as much as the Universities have tried to adapt the medical model and teach it to you it’s not the same thing (Pilot EC ACP 14)

### EC ACP training needs some flexibility to reflect the individual—‘one size does not fit all’

Respondents highlighted variation in pre-existing knowledge, skills and personal circumstances of trainee EC ACPs at training outset (partly due to ACPs being recruited from a variety of healthcare professional backgrounds), with significant life events (eg, illness, caring responsibilities) also impacting on training progression. Respondents felt that decisions about RCEM credentialing targets could be made on a case-by-case basis, so that individual experience and circumstances could be considered in preference to a ‘one size fits all’ approach to training.

I think it’s really difficult to actually say “you should be achieving this, this, and this within this timeframe”, because you know, your baseline knowledge, and understanding, happiness for the context, previous educational experience, all of that, like your family life, all of that comes into play, and has a big impact on what you’re able to do, and what your most challenging parts will be (Consultant EC ACP lead 5).

It’s clear that people progress at different speeds… you can say you’ll take three years from arrival to credentialing, but some may take five, but some may be ready in two (Consultant EC ACP lead 3).

Perceived workload pressure was higher for those who were undertaking their master’s in advanced practice alongside completion of the RCEM credentialling e-portfolio than for those who had completed the master’s prior to commencing ACP training. Trainees who undertook the master’s and RCEM credentialling e-portfolio simultaneously described feeling at a disadvantage and the workload as relentless.

The modules kind of come pretty quickly in the back, back-to-back, you don’t really have any let ups from the University side of things and like I say your SPA time gets eaten by University time that then doesn’t give you time to concentrate on your portfolio, then you end up in this cycle of, of getting nowhere fast, all being sort of overwhelmed with workload … you can’t continue to work at 120% all the time… I don’t know how long that’ll be sustainable for (Pilot EC ACP 11)

So I think having got most of the academic stuff under my belt already gave me an advantage as it meant I could focus much more of my time specifically on getting sort of my practical stuff, my clinical knowledge, and my portfolio sorted (Non-pilot EC ACP 4).

### Supervision and teaching requires significant staff capacity that is often impacted by external pressures

Although trainee EC ACPs were assigned an educational supervisor (either a consultant or other senior clinician) who was responsible for the management of their educational progress during training, most of the workplace-based clinical learning was described as resulting from informal and unplanned interactions with medical colleagues within the ED. Trainee EC ACPs explained medical colleagues would constructively challenge their clinical thinking, something which they had not experienced while working in their baseline profession (eg, nurse or paramedic). This was viewed positively and was felt to create a supportive learning experience:

For ten years no one ever stepped foot in the ambulance and saw me assess a patient… whereas in medicine obviously it’s a constant cycle of being assessed by your peers and getting feedback … you’re in this constant feedback loop which is completely new to us, but it makes you feel really supported compared to where we’ve all come from previously (Pilot EC ACP 2)

It was a requirement that all trainee EC ACPs had to have every patient either physically reviewed by a senior ED clinician or at least have a clinical conversation before the patient could be discharged. At the start of their training, some trainee EC ACPs struggled with this loss of autonomy, with one trainee EC ACP saying: *I felt initially like my like my wings were being clipped (Pilot EC ACP 8)*. However, most trainee EC ACPs valued the additional support provided by senior ED clinicians, using them as a safety net, because *when you step into the role you don’t know what you don’t know (Pilot EC ACP 7)*.

Formal supervision requirements were difficult to meet with both trainee EC ACP and consultant EC ACP leads recognising problems in providing appropriate supervision and signing off workplace-based assessments for RCEM credentialling e-portfolios while EDs were under significant pressure.

If you’re staffing fifty ambulances and the department looks like it’s on fire and it’s you know, it’s quite hard to then amend supervision for some of those who are working reasonably slowly and needs all of their, their work double checked, and patients re-examined. So it’s not insurmountable and the policy is very good. So I’m not concerned that they don’t have that supervision (Consultant EC ACP 4)

So in 10 minutes when you’re set up about to do a procedure and they don’t turn up because there’s something else that’s unfolded, you’re not gonna delay that patient’s care, you’re gonna continue doing the procedures … But at the same time it’s very disheartening that I’ve had this perfect opportunity and there’s no one to see it (Pilot EC ACP 10).

Having to keep going and begging consultants can you please sign this off … It’s just really frustrating cause you feel bad keeping going round and begging people for things and this is what I feel like I’m doing all the time (Pilot EC ACP 14)

Consultant EC ACP leads described having to balance the training needs of trainee EC ACPs alongside those of junior doctors to ensure everyone gets equitable access to learning opportunities, perceiving EC ACPS as having higher training and supervision needs. Sharing the supervision workload throughout the team was described as beneficial. Some departments participated in an RCEM educator pilot where funding was provided by HEE enabling consultants to have ‘protected time’ to deliver workplace-based teaching and supervision for all ED trainees (not just EC ACPs) in the department.

I would definitely recommend a team of consultant supervisors with time aside for supervision from the get-go and understanding that it’s going to be energy and time-intensive…We have introduced daily educator shifts which are 4-hour educator slots so that the consultant is not clinical in as much as they’re not on the numbers … they can supervise procedures, they can do workplace based assessments, they can do, you know, one-to-one teaching, they can do simulation training, they can do all those sorts of stuff. I think that’s been helpful (Consultant EC ACP 5)

### Unclear role expectations and responsibilities hinder role transition and impact role identity

There was consensus among the trainee EC ACPs that the transition from their baseline profession (nurse or paramedic) to trainee EC ACP was challenging. One trainee EC ACP said they felt like they had been: *Drop kicked into the fire (Pilot EC ACP 13),* with another stating *It’s probably the most extreme kind of process I've ever been a part of (Pilot EC ACP 10)*.

Difficulties transitioning into ACP training were exacerbated due to unclear role expectations. While trainee EC ACPs described feeling like they were embedded within the medical team in terms of their supervision and training, there was still confusion about where the EC ACP role fitted within the wider ED team, contributing to feelings of isolation.

I’d gone right back to square one again, I wasn’t part of the doctors, I wasn’t part of the nurses, I didn’t really know where I fit … it was just a difficult transition (Pilot EC ACP 10).

Because the ACP role is still quite a new role, people still don’t really know where we sit so are we team nurse or are we team doctor? (Pilot EC ACP 5)

Trainee EC ACPs were keen to develop a unique identity, which complements but does not replace the doctor’s role. However, a lack of clear role identity for trainee EC ACPs sometimes meant a reluctance to fully embrace a transition from their baseline profession, often describing themselves as a nurse or paramedic with extended skills rather than an ‘ACP’.

I still refer to myself as a paramedic … I still see myself in that role but with the ACP attachment to it (Pilot EC ACP 8).

I just know that I'm not a doctor (Pilot EC ACP 7)

When departmental pressures were high, trainee EC ACPs reported slipping back into roles associated with their baseline profession to ‘help’ their nursing colleagues, which was sometimes viewed negatively by their medical colleagues. However, some trainee EC ACPs described this as a unique feature of the ACP role.

The benefit of the ACPs is having an all-round clinician that can do everything for the patients starting from triage to discharge. But I feel that maybe as a department we missed the mark a little bit on that … We are very much aligned with the, the doctors it’s all about clerking the patients, and the rest of the bits and pieces of the department don’t really come into play. So, we, we have almost moved away from the nursing side of things … whereas I think like that’s something fundamental to the role really (Pilot EC ACP 11).

Trainee EC ACPs described the importance of having access to a strong support network to help overcome transitional anxieties, including people who had been through the ACP training process who can relate to the unique challenges experienced by trainee EC ACPs.

Just knowing that the other people that are training feel the same way as you or like you know you do something and you feel like an absolute idiot and then the next thing they text you and say oh my god you'll never guess what I did and that makes you feel a bit better (Pilot EC ACP 3).

I've been listening to some podcasts recently, whereby other ACPs, consultant ACPs have shared their journey and their experience. And it’s been incredibly powerful … to hear those people up ahead, say, do you know what, I finished my masters, I finished my RCEM credentialing. And it took me two, possibly three years before I actually felt comfortable with what I was doing. And it was like an epiphany (Pilot EC ACP 10)

We definitely look at things differently I’d say to doctors and so it’s nice to be able to sometimes discuss things with your team of ACPs (Non-pilot EC ACP 3).

## Discussion

Eighteen trainee EC ACPs and five consultant EC ACP leads were interviewed in this qualitative study. Four themes were identified from the data: (1) the master’s in advanced practice could be better aligned with the RCEM credentialling e-portfolio; (2) a recognition that EC ACP training needs some flexibility to reflect the individual—‘one size does not fit all’; (3) supervision and teaching is recognised as important and highly valued by all levels of staff, however it requires significant staff capacity that is often impacted by external pressures; (4) unclear role expectations and responsibilities hinder role transition and impact role identity. These themes will now be discussed within the context of the existing literature.

Previous literature identified the importance of a clear supervision structure within ACP training.^10^,^15^ Trainee EC ACPs participating in the HEE SW pilot received a significant amount of supervision and support from colleagues within the ED. This contrasts to a national survey, which found only 32% of ACPs said they had a formal structure for their supervision.^1^ However, trainee EC ACPs in our study said increased ED pressures reduced ED staff capacity to conduct workplace-based clinical assessments. These issues are not unique to trainee EC ACPs and have been shown to affect other trainees (eg, junior doctors).^16^,^18^ To resolve these issues, some ED teams had implemented daily educator shifts (as part of an RCEM educator pilot), where consultants had protected time to deliver training and supervision for all ED trainees (not just EC ACPs).

Evidence from this evaluation, and previous literature,^10 19^ highlights the variability in pre-existing skills and knowledge of staff entering ACP training programmes, affecting their rate of progression and confidence in the role. Standardised recruitment criteria may help reduce the variation in the pre-existing skills of EC ACPs at the start of training. Additionally, while timelines for completion of RCEM credentialing are important to ensure trainees do not stay in a training capacity indefinitely, an element of flexibility, which takes into consideration individualised learning needs and significant life events could be useful.

In our evaluation, concerns were raised about a disconnect between the workplace-based clinical training (RCEM credentialling e-portfolio) and academic elements of ACP training (master’s in advanced practice), with the latter being described as not sufficiently clinically focused enough to help trainee EC ACPs in their workplace-based clinical training. The master’s in advanced practice teaches generic clinical skills to all ACPs regardless of specialty. Due to the wide range of clinical settings ACPs are working, it is likely be challenging to include specialty-specific modules within a 3-year master’s programme. Therefore, as part of the RCEM credentialling process, EC ACPs could be provided with additional academic training in topics that are not able to be covered by the generic master’s in advanced practice.

Interestingly, while trainee EC ACPs talked about increasing their skills regarding the clinical pillar of the multiprofessional framework,^6^ the other three pillars (leadership and management, education and research) were not discussed or when they were discussed those elements were described as a ‘tick box exercise’ they had to complete for their master’s in advanced practice rather than being an integral part of their ACP training. A similar observation has been reported in the previous literature.^10 11 20^ Overwhelming pressures on ED services understandably means seeing patients has become the top priority for EC ACPs. However, it does mean that their capabilities and potential within the other three pillars of the multiprofessional framework could be overlooked.

We identified a lack of clear role identity for ACPs, an issue identified in previous literature.^111 12 20^,^22^ The multiprofessional framework for advanced practice was designed to provide more clarity around the ACP role.^4^ Furthermore, at the end of the first year of the HEE SW ACP pilot (August 2020), the national landscape in England regarding ACP training and integration into workforce planning changed, with the establishment of the HEE Centre for Advancing Practice. The HEE Centre for Advancing Practice works with individual providers and Integrated Care Boards (an NHS organisation responsible for arranging the provision of health services within a geographical area) to ensure advanced practice training and roles are better understood, planned for and supported across regions and in the workplace. The Centre also works with education providers to ensure that the MSC in Advanced Practice courses meet set standards. However, despite these changes, trainee EC ACPs interviewed in our evaluation still struggled to define the ACP role. It is possible that it is too early to assess the impact of the multiprofessional framework and subsequent changes to advanced practice training (eg, the establishment of the HEE Advance Practice Centre) on role identity issues.

As ACP teams continue to grow and become more embedded within multidisciplinary teams, issues around role identity may resolve as awareness of their scope of practice increases. Further research should assess the impact of the multi-professional framework and training programmes on role identity issues in the long term.

### Limitations

The evaluation was undertaken during the COVID-19 pandemic, which undoubtedly had an impact on the implementation of the HEE SW pilot. For example, some trainees experienced a delay to the start of the master’s in advanced practice, with most modules taking place online; face-to-face regional support days organised by HEE SW were unable to go ahead as planned; and opportunities to attend workplace-based clinical placements outside of the ED were cancelled. This means the benefits of the HEE SW pilot may not be fully realised. However, many of the issues raised in this evaluation, such as exponential pressures on EDs and how that impacts the training experience, precede the COVID-19 pandemic and should be taken into consideration when developing future guidance around ACP training in emergency care.

## Conclusion

Increasing demands on ED services can limit staff availability to provide workplace-based training and clinical assessment opportunities, impacting the rate of progression through EC ACP training. It was notable that EC ACPs primarily spoke about the development of their clinical skills despite there being other skills mentioned in the multiprofessional framework for advanced practice (leadership and management, education and research). ACP role identity issues continue to persist despite the introduction of new guidance (eg, Multidisciplinary Framework for Advanced Clinical Practice) designed to create a shared understanding of what advanced practice entails.

## supplementary material

10.1136/emermed-2024-214016 online supplemental file 1

10.1136/emermed-2024-214016 online supplemental file 2

## Data Availability

Data are available upon reasonable request.
