# Supplementary material for: A qualitative study exploring the experiences of advanced clinical practitioner training in emergency care in the South West of England, United Kingdom
Source: Emerg Med J. 2024 Oct 15;42(3):e214016. doi: 10.1136/emermed-2024-214016 (PMC11874414; doi:10.1136/emermed-2024-214016)
Supplement: online supplemental file 1 [file emermed-42-3-s001.pdf]

# Draft interview schedule – Trainee Emergency Care ACPs

**Opening question**

1. Please can you briefly tell me about your past work experience and why you decided to enrol on the emergency care ACP training programme? [Need to specify whether pilot-training programme or non-pilot]

**Experiences of ACP training**

2. Please can you describe your overall experience of ACP training?

    - Probe: Support and supervision / clinical and external educational opportunities / peer support and support from other colleagues / work placements (positive and negative aspects)
    - Probe: If negative aspects highlighted, ask them what could be done to improve this situation.

3. Do you have clear planned goals and objectives within your training?

    - Probe: Are these what they expected them to be as a trainee ACP?

4. Are there any gaps in your training that you feel should be addressed?

**Areas for improvement**

5. Is there anything that could be done to improve training for ACPs?

**Career progression**

6. What are your thoughts on career progression within the ACP role?

**Role identity**

7. How have you found moving from an Allied Health Professional role to a medical model?
    - Probe: Support making this transition

8. What do you perceive the ACP role being? Do you think it will change? If so, how?

9. How do other people within your organisation perceive the ACP role?

**Closing question**

10. Is there anything else that you would like to discuss with regards to your experiences of ACP training or ACP training in general?
