# Supplementary material for: A qualitative study exploring the experiences of advanced clinical practitioner training in emergency care in the South West of England, United Kingdom
Source: Emerg Med J. 2024 Oct 15;42(3):e214016. doi: 10.1136/emermed-2024-214016 (PMC11874414; doi:10.1136/emermed-2024-214016)
Supplement: online supplemental file 2 [file emermed-42-3-s002.pdf]

Draft interview schedule – Strategic Leads

Opening questions

1. Please can you briefly describe your current role within [name of organisation]?

2. What involvement have you had in the new pilot ACP emergency care training programme?

Comparison between existing training and new pilot ACP training programme

3. Please can you briefly describe what ACP training was like in your department prior to the implementation of the new pilot ACP emergency care training programme?

4. Have you noticed any differences between the new pilot ACP emergency care training programme and existing ACP training programmes?

    - Probe: elements that are better or worse than the existing training programme. Things working well and areas that could be improved.

Impact of new ACP training programme on other staff

5. Has the new pilot ACP emergency care training programme had an impact on other staff within the department?

    - Probe: trainee ACPs / other healthcare staff / trainers
    - Probe: Positives and negatives
    - Probe: role conflicts / increased workload / better awareness about ACP role

Future planning

6. Do you see a future for ACPs working in your department? If yes, how do you see the ACP role working in your service in the future?

    - Probe: ongoing CPD / career development / role identity

7. Are there any changes you would like to make to the new emergency care ACP training programme?

Closing question

8. Is there anything else you would like to discuss with regards to the new pilot ACP training programme or ACP training in general?
